# Successful Treatment of Syphilis

**Published:** 1888-05

**Authors:** George Howe

**Affiliations:** New Orleans, Louisiana


### SUCCESSFUL TREATMENT OF SYPHILIS.

BY GEORGE HOWE, M. D., NEW ORLEANS, LOUISIANA.

The results of another year's use of succus alterans finds me, if possible, a more enthusiastic advocate of its use in all stages of syphilis.

Among those cases which have come under my care are two which may be of more than usual interest.

Mr. B. C— came to New Orleans in December, 1886, to attend to some business which kept him here about three months. He had been suffering from syphilis for nearly six years and had been under the care of eminent practitioners in his native State for a long period. Changing his residence to another State, he was again under medical care. He had been repeatedly mercurialized, iodized and in fact had gone through a regimen of treatment and diet which was heroic in the extreme. Among other remedies iodide of potassium had been administered until he was taking the enormous amount of seven hundred grains daily. He is of more than ordinary intelligence and had kept a record of the doses and their gradual increase until the amount, a little exceeding seven hundred grains daily, was taken for some time.

When he came to me his condition was such as would have enlisted the sympathy of any one. About five feet eight inches in height, and weighing one hundred and fifteen pounds—a countenance expressive of suffering and the utmost resignation to a life of continued torture—impaired digestion by day and osteocopic agony at night—in fact, the most unpromising case I had ever seen.

With some difficulty he was persuaded to use succus alterans in a methodical manner and at once began to take it in ℨii doses three times daily. A few days after, he returned and desired some relief from the pains, nocturnal and diurnal, caused by nodes in formation and those already fully developed.

He was advised to use a four (4) per cent. solution of cocaine. Hydrochlorate, painted over each seat of pain and half an hour after its application to paint the same surface with tinct. iodine. Immediate relief followed, and it was not necessary to use it more than once during the night, except upon one occasion. This treatment gave such relief as to permit its being discontinued after about ten days. He was also advised to use fifteen grains potas. iodide during the day, taking five grains with each dose of succus. This was followed for one month, then the potas. iodide was dropped and the treatment confined to succus alone. At the end of the second month he began the maximum ℥ss dose (Feb., 1887) and kept it up till September, 1887, then at my suggestion reduced the dose ʒi three times daily.

About November 20th, 1887, he returned to New Orleans and called on me. I did not recognize him in his improved appearance. Has never suffered from the osteocopic pains since nearly eleven months, has increased in weight and his complexion and general appearance indicate a return to health. He will continue succus alterans for six months longer in ℥ss doses twice daily for two months, then very gradually reduce to ʒi twice daily. This case alone has been so complete a success that I do not hesitate to advise succus alterans in all stages of syphilis.

Mr. G—, an Israelite of this city, came under my care in December, 1886, then in the secondary stage, with characteristic eruption, sore throat and alopecia. Had been thoroughly salivated, and, thus disgusted with that course of treatment, was at once ordered succus alterans in ʒi doses three times daily for two weeks, then increased to ʒii doses for another two weeks, then ʒiii doses for one month, then ℥ss doses three times daily, which was continued for about four months, and with such complete success that I reduced the doses one-half. While taking this reduced dose he again contracted syphilis—two chancres, followed by fever eruption, sore throat and loss of hair, thus demonstrating his complete cure so far as first poisoning was concerned, as it is now an accepted fact that no one suffering from syphilis can be re-inoculated with the same poison. Again the treatment was resumed and the course fully carried out with a complete cure in about six months.

I notice the difficulty attending the treatment of the Israelite suffering from syphilis. A number of cases have come under my care, and I find them to be particularly obstinate and much less likely to be relieved in a specified time than the Christian. Is this the result of intermarriage, with a highly developed heriditary taint, or the result of their mode of living? I would like to have the views and experience of other physicians of this.

Other cases, possessing no distinctive features, have been treated with unusual success, and in no case have I found recourse to mercury necessary or advisable to hasten cure.

